# Imaging of lower extremity infections: predisposing conditions, atypical infections, mimics, and differentiating features

**DOI:** 10.1007/s00256-024-04589-4

**Published:** 2024-01-19

**Authors:** George R. Matcuk, Sanaz Katal, Ali Gholamrezanezhad, Paolo Spinnato, Leah E. Waldman, Brandon K. K. Fields, Dakshesh B. Patel, Matthew R. Skalski

**Affiliations:** 1 Department of Imaging, Cedars-Sinai Medical Center, Los Angeles, CA 90048 USA; 2 Melbourne, Australia; 3 Department of Radiology, Keck School of Medicine, University of Southern California, Los Angeles, CA 90033 USA; 4 Diagnostic and Interventional Radiology, IRCCS Istituto Ortopedico Rizzoli, 40136 Bologna, Italy; 5 Department of Radiology, Duke University School of Medicine, Durham, NC 27705 USA; 6 Department of Radiology & Biomedical Imaging, University of California, San Francisco, San Francisco, CA 94143 USA; 7 Department of Radiology, Palmer College of Chiropractic—West Campus, San Jose, CA 95134 USA

**Keywords:** Diabetes mellitus, Peripheral arterial disease, Neuropathic arthropathy, Atypical infection, Mimics, Imaging

## Abstract

Imaging evaluation for lower extremity infections can be complicated, especially in the setting of underlying conditions and with atypical infections. Predisposing conditions are discussed, including diabetes mellitus, peripheral arterial disease, neuropathic arthropathy, and intravenous drug abuse, as well as differentiating features of infectious versus non-infectious disease. Atypical infections such as viral, mycobacterial, fungal, and parasitic infections and their imaging features are also reviewed. Potential mimics of lower extremity infection including chronic nonbacterial osteomyelitis, foreign body granuloma, gout, inflammatory arthropathies, lymphedema, and Morel-Lavallée lesions, and their differentiating features are also explored.

## Introduction

Imaging plays an important role in the diagnosis of lower extremity infections. However, this can be complicated in the setting of underlying conditions or with atypical infections. This review presents the imaging features of underlying predisposing conditions, such as diabetes mellitus, peripheral arterial disease, and neuropathic arthropathy. The imaging manifestations of atypical infections, including viral, mycobacterial, fungal, and parasitic infections, are also examined. Potential mimics of lower extremity infection such as chronic nonbacterial osteomyelitis, foreign body granuloma, gout, inflammatory arthropathies, lymphedema, and Morel-Lavallée lesions will be discussed, and their differentiating features highlighted.

## Predisposing conditions

There are a number of underlying conditions that may predispose to and have overlapping features with lower extremity infection, including diabetes mellitus, peripheral arterial disease, and neuropathic arthropathy [1]. Peripheral arterial disease and neuropathic arthropathy often occur secondary to diabetes mellitus but can also be due to other etiologies.

### Diabetes mellitus

Thirty seven million three hundred thousand people in the USA have diabetes, and another 96.1 million are considered prediabetic [2]. Diabetes mellitus predisposes to lower extremity infections through several pathological factors, including immunological dysfunction, vascular insufficiency, and neuropathy [3]. Hyperglycemia causes defective neutrophil function with disruption of migration patterns and reduced production of chemotactic factors, which impairs the immunological response to infection [4]. Hyperglycemia also increases osmotic stress in vessels and reduces nitric oxide levels, stimulating vasoconstriction. Arteriosclerosis, alteration of vascular cell apoptosis and proliferation, impaired hemostasis, increased vascular permeability, and excessive leukocyte adhesion also contribute to diabetic vascular insufficiency [5]. Diabetic neuropathy affects motor, sensory, and autonomic nerves, with motor and sensory damage leading to muscle atrophy and foot deformity (Charcot arthropathy) resulting in biomechanical changes that predispose to inadvertent foot trauma. Autonomic neuropathy causes vasodilation with erythema and impaired control of sweat glands, leading to anhidrosis with dry skin which is prone to fissuring and callus formation [4]. This combination of factors predisposes to ulceration, impaired healing, and lower extremity infection in diabetic patients. The most common infecting microorganism is *Staphylococcus aureus*, with methicillin-resistant (MRSA) strains comprising up to 17–30% of diabetic foot infections [6].

Diabetic myopathy is a complex interplay of changes in muscle metabolism due to chronic hyperglycemia and secondary changes due to motor neuropathy and vascular insufficiency [7, 8]. This can lead to generalized edema (T2 hyperintensity) with eventual fatty atrophy of the intrinsic foot and even the calf musculature on MRI [9]. It is important to differentiate from diabetic muscle infarct or myonecrosis, which will also demonstrate intramuscular iso-or decreased T1 and increased T2 signal intensity, but will also have muscle enlargement, with areas of near fluid signal intensity necrosis demonstrating rim enhancement on post-contrast images [10]. On ultrasound, diabetic muscle infarctions appear as well-marginated, hypoechoic intramuscular lesions with internal linear striations, lack a predominantly anechoic region, and do not show swirling fluid with transductor pressure [11]. CT reveals a low-attenuation intramuscular lesion with ring enhancement. There may be adjacent perifascial fluid and overlying subcutaneous edema.

**Fig. 1:**
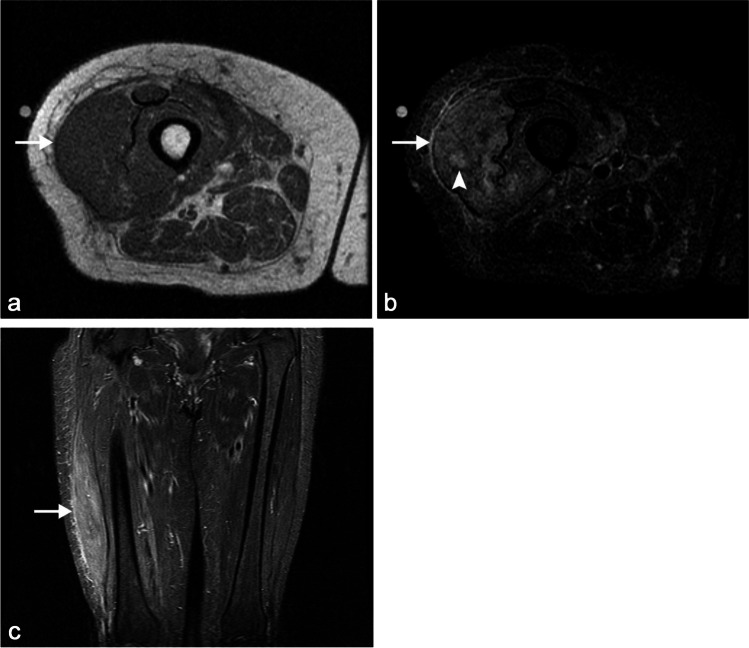
Fifty-five-year-old woman with poorly controlled type 2 diabetes mellitus with right thigh pain. Axial T1 (**a**), STIR (**b**), and coronal STIR (**c**) MR images demonstrate diffuse swelling and edema of the right vastus lateralis muscle (*arrows*) with a small focus of myonecrosis (*arrowhead*, **b**). The patient had no fever or leukocytosis and responded well to conservative management, differentiating this patient’s diagnosis of diabetic muscle infarct from pyomyositis, which can otherwise look similar on imaging

Diabetic muscle infarct or myonecrosis is a rare complication of long-standing insulin-dependent diabetes and is usually a self-limiting disease that typically responds well to conservative management. It most often involves the thigh (usually quadriceps) muscles but may also involve the calf or rarely the upper extremity muscles (Fig. 1). Myonecrosis can also be seen in cancer patients secondary to radiotherapy or other treatment [12]. Involvement of the foot muscles has not been reported, in comparison to diabetic myopathy, where foot involvement is often most severe.

Diabetic muscle infarct and myonecrosis may be difficult to differentiate from pyomyositis, intramuscular abscess, and necrotizing fasciitis on imaging, although with pyomyositis, the muscle inflammation tends to be more diffuse and intense; with intramuscular abscess, the rim enhancement is more thick and irregular, and with necrotizing fasciitis, the deep perifascial fluid and enhancement are more extensive and may potentially show soft tissue gas [13]. Clinical information is also important for differentiating infection from diabetic myopathy or muscle infarct, including the presence of systemic symptoms such as fever and leukocytosis.

### Peripheral arterial disease

**Fig. 2:**
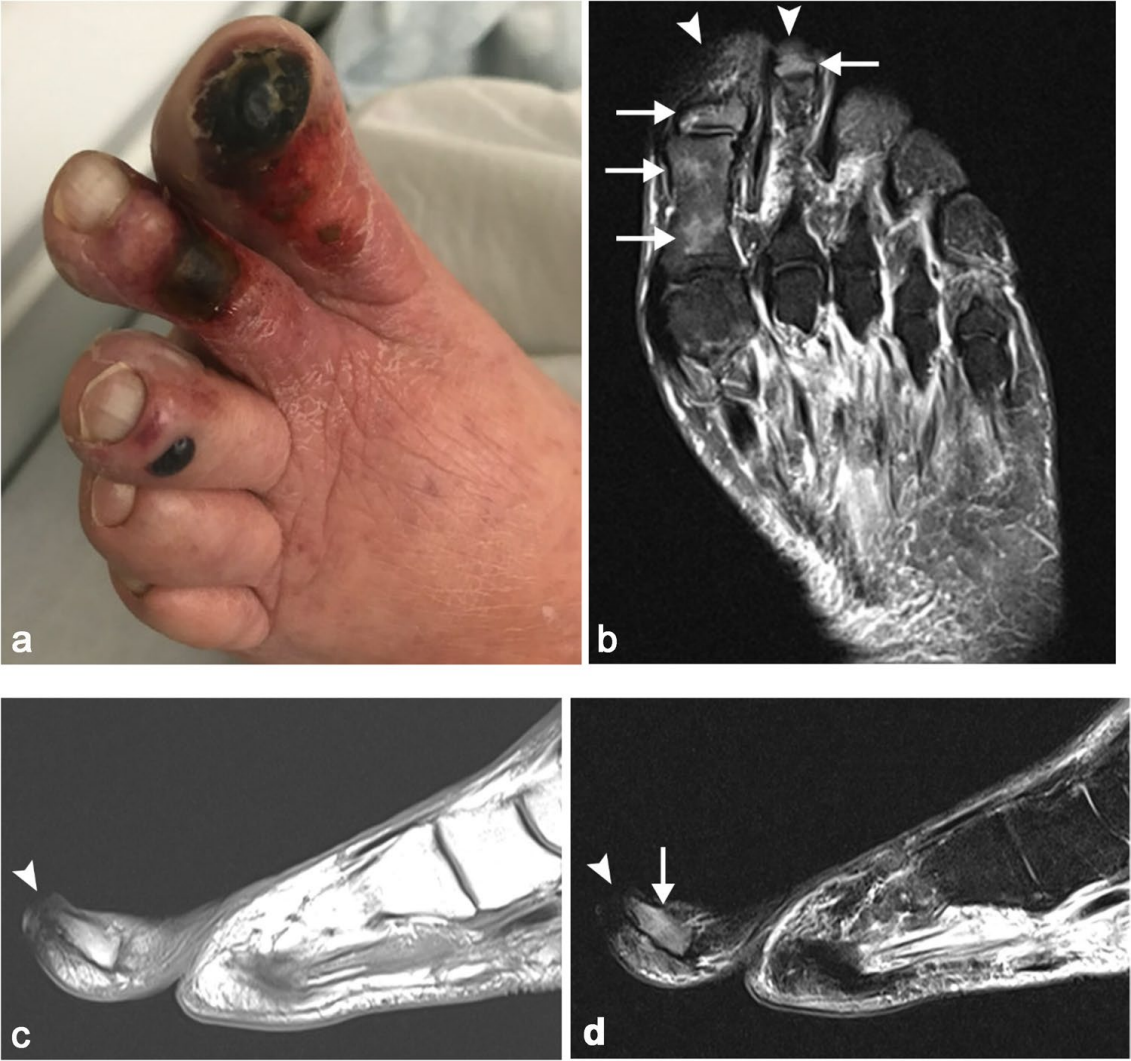
Eighty-five-year-old man with peripheral arterial disease with worsening pain and discoloration of his left toes. Clinical photograph (**a**) demonstrates areas of gangrene of his first, second, and third toes. Long-axis STIR (**b**), sagittal T1 (**c**), and STIR (**d**) MR images demonstrate signal loss of the soft tissues in the areas of gangrene (*arrowheads*) with patchy STIR hyperintensity (*arrows*) but normal T1 signal intensity within portions of the first proximal and distal and second distal phalanges, compatible with vascular insufficiency without superimposed infection (dry gangrene)

Peripheral arterial disease and pedal ischemia can result from a combination of macrovascular and microvascular disease [14]. Macrovascular disease can be evaluated with conventional, CT, or MR angiography. Early soft tissue ischemia may have a normal appearance on T1 and T2 MR images or show subtle edema or slight decreased enhancement relative to adjacent non-ischemic areas. As ischemia progresses to devitalization (non-infected or dry gangrene), there is regional soft tissue loss (more pronounced on STIR compared to T1 sequences, Fig. 2) and absence of post-contrast enhancement. There may be superimposed foci of signal void within devitalized areas corresponding to gas, which is usually due to overlying skin ulceration/fissuring and does not generally imply infection with a gas-forming organism (gas gangrene). Bone infarction may also be present in ischemic areas and is characterized by a serpentine pattern with well-defined margin and adjacent bone marrow edema.

Superimposed infection of devitalized tissues (wet gangrene) will show the typical replacement of subcutaneous (cellulitis) or bone marrow (osteomyelitis) fat signal intensity with T1 hypointensity and T2 hyperintensity on pre-contrast images, but it is important to note that post-contrast enhancement will still be absent in these devitalized areas, even if infected. Similarly, soft tissue abscesses in devitalized tissue will appear as a focal fluid signal intensity collection but will not demonstrate rim enhancement.

### Neuropathic arthropathy

Neuropathic osteoarthropathy (Charcot foot) was first described in patients with tabes dorsalis (syphilis) by Jean-Martin Charcot in 1868, but is most commonly secondary to diabetes [15, 16]. Other causes include spinal cord lesions (including syringomyelia, tumors, meningomyelocele, extrinsic compression, multiple sclerosis, and poliomyelitis), alcoholism, uremia, amyloidosis, pernicious anemia, congenital insensitivity to pain, familial dysautonomia (Riley-Day syndrome), hereditary sensory and motor neuropathy (Charcot-Marie-Tooth disease), and leprosy [16]. There are two main theories of etiology: the neurotraumatic theory, where absence of normal protective sensory feedback leads to repetitive unperceived trauma; and the neurovascular theory, where autonomic dysfunction causes vasodilation and hyperemia with resultant osteopenia, bone resorption, and fracture [17].

In the lower extremity, neuropathic arthropathy typically presents with a hypertrophic pattern which in the chronic stage is characterized by the “6 D’s” on plain radiographs: joint Distension (effusions), Destruction, Dislocation, Disorganization, Debris, and increased bone Density (relative sclerosis) [18]. The atrophic form with dominance of bone resorption that may mimic septic arthritis is more common in the non-weight-bearing joints of the upper extremity [17]. The destructive changes of the hypertrophic form can be difficult to distinguish from superimposed septic arthritis and osteomyelitis on imaging, particularly on plain radiographs and CT, and even clinically.

**Fig. 3:**
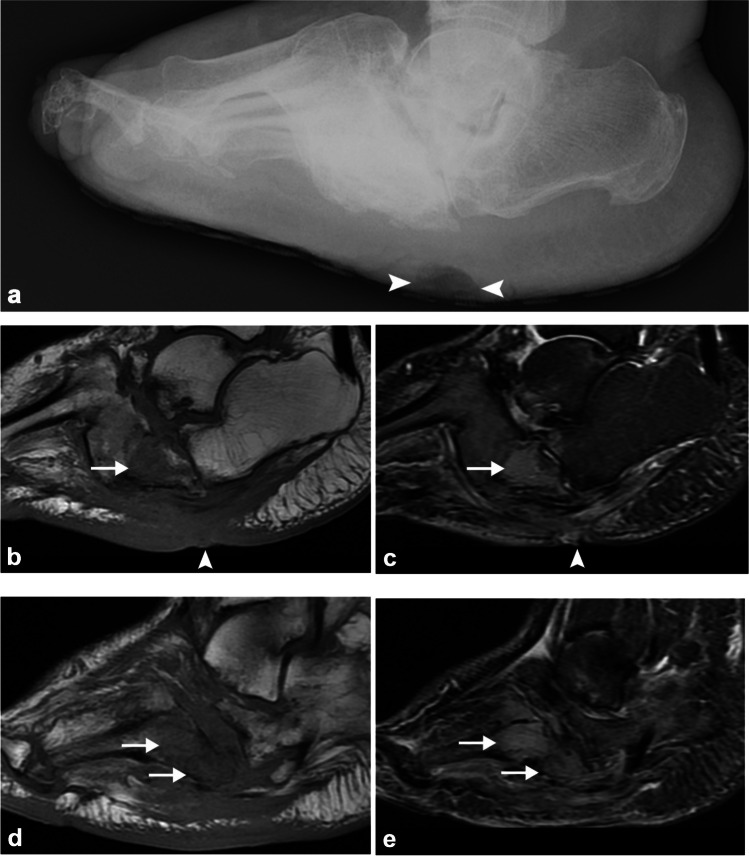
Fifty-six-year-old diabetic man with left foot ulcer and soft tissue swelling. Lateral radiograph (**a**) demonstrates disorganization, relative increased density, dislocations, and rocker-bottom deformity of the midfoot, compatible with neuropathic arthropathy (Charcot foot) with a plantar soft tissue ulcer (*arrowhead*). Sagittal T1 (**b**) and STIR (**c**) MR images at the level of the cuboid and sagittal T1 (**d**) and STIR (**e**) MR images at the level of the fourth and fifth metatarsal bases demonstrate bone marrow replacement with increased STIR and decreased T1 signal intensity in the cuboid and proximal fourth and fifth metatarsal bases (*arrows*) compatible with superimposed osteomyelitis subjacent to the ulcer

When superimposed infection is suspected, MRI is the preferred modality for evaluation. MRI features that are helpful for diagnosing superimposed infection and osteomyelitis include overly soft tissue changes such as ulcer, abscess, tenosynovitis, or sinus tract; typical location of involvement such as toes and weight-bearing regions such as metatarsal heads and calcaneus; and pattern and distribution of MRI signal change [19]. With neuropathic arthropathy, the bone marrow edema pattern tends to be periarticular, subchondral (although advanced cases may demonstrate diffuse T1 hypointensity and T2 hyperintensity, mimicking osteomyelitis), and involving several bones/joints, whereas superimposed osteomyelitis tends to be more focal, sometimes affecting only a single bone, with diffuse bone marrow involvement (Fig. 3).

Post-contrast imaging may be helpful for demonstrating superimposed osteomyelitis, particularly when there is low T1 signal on pre-contrast images with poor definition of the bones (“disappeared”) that then “reappear” after contrast administration, also known as the “ghost sign” [20]. Advanced techniques such as diffusion-weighted imaging (osteomyelitis demonstrates restricted diffusion while bone marrow edema does not) and dynamic contrast enhancement (DCE) (which may show different perfusion patterns between osteomyelitis and osteoarthropathy) may increase specificity [21, 22].

However, there remains some overlap in the MRI appearance of neuropathic arthropathy with and without infection. In non-conclusive cases, nuclear medicine imaging including white blood cell scintigraphy and 18F-FDG PET (or PET-CT) may be helpful, with a higher specificity for superimposed osteomyelitis [23]. For scintigraphy, it may be useful to combine leukocyte scintigraphy with bone scintigraphy and sulfur colloid bone marrow imaging (a triple tracer study), which improves diagnostic accuracy for osteomyelitis in the diabetic foot, with a sensitivity of 92% and specificity of 100% [24].

### Intravenous drug abuse (IVDA)

It is estimated that approximately 16 million people worldwide inject illicit drugs on a regular basis, including over 600,000 Americans, with heroin the most commonly injected drug [25]. Approximately one-fifth of intravenous drug abusers inject the foot, making it the fourth most common site of injection in the body [26]. Injection of the lower extremities can result in chronic venous insufficiency (13%), leg ulcers (10%), and deep venous thrombosis (23%) [27]. Long-term IVDA can result in sclerosis of veins and lead some user to inject subdermally, a practice also known as “skin popping” [25]. Approximately one-fourth of patients with IVDA develop cellulitis or abscess, with the incidence rising to 61% in skin poppers. These soft tissue infections can lead to ulcerations, which some abusers maintain as a “shooter’s patch,” using the granulation tissue as another means for injecting illicit drugs. Most infections are caused by *S. aureus*; some infections can progress osteomyelitis. Most infections require some form of surgical debridement; however, it can be challenging to ensure that these patients get the appropriate care, with 12% of patient leaving against medical advice and nearly three-fourths not returning for scheduled outpatient visits [28].

## Atypical infections

Most lower extremity infections are bacterial in origin, most commonly Staphylococcus and Streptococcus species, and may be polymicrobial, with aerobic and anaerobic bacteria present at the same time [29]. However, atypical bacterial, fungal, viral, and parasitic infections can also be causes of lower extremity infections in both immunocompetent and immunocompromised patients, with a wide range of imaging appearances [30].

### Viral infection

Many viral infections can cause skin rashes, myalgia or myositis, and arthralgias. Despite clinical presentations which include joint effusion, swelling, and synovitis [30], imaging findings may be subtle or inapparent. Viral osteomyelitis mostly occurs in neonates and children, with radiolucent metaphyseal lesions and periarticular osteopenia adjacent to the growth plate. Congenital rubella classically produces longitudinal striated sclerotic bands at the metaphysis giving a celery stalk appearance.

Chikungunya virus is a mosquito-borne arbovirus in tropical regions that can present with fever, rash, and debilitating polyarthralgia that can last for months following infection [31]. Joint involvement most often affects the hands, wrists, ankles, and metatarsophalangeal joints [32]. On radiography, joint space narrowing (57%) and erosions (24%) can be seen 10–18 months post infection, with up to 81% demonstrating these findings at 24 months. MRI is more sensitive for erosions, which can be seen as early as 6 months after infection with this modality [33]. MRI findings can mimic rheumatoid arthritis with periosteal inflammation, bone marrow edema, joint effusions, synovitis, and tendinitis/tenosynovitis [34].

COVID-19 infection can likewise present with musculoskeletal manifestations, including myalgia, myositis and rhabdomyolysis (with muscle edema or myonecrosis on MRI), peripheral neuropathy (with nerve enlargement and loss of fascicular architecture on US or MRI, and T2 hyperintensity on MRI with or without muscle denervation edema), arthralgias (with joint effusion and synovitis on US and MRI), osteopenia and osteonecrosis (probably secondary to corticosteroid therapy and/or hypercoagulability), intramuscular hematomas (due to frequent anticoagulation use to prevent or treat COVID-19-related thromboembolic disease), atypical decubitus ulcers (related to prolonged hospital stays and prone positioning), and gangrene (“COVID toes”) [35, 36]. Proposed mechanisms of these COVID-19 musculoskeletal manifestations include systemic inflammatory dysregulation (cytokine storm), prothrombic state (hypercoagulability), and autoimmunity [37]. Up to 10–20% of patients will develop long- or Post-COVID-19 condition with symptoms of fatigue, shortness of breath, arthralgias, muscle pain or spasms, and post-exertional malaise due to long-standing COVID-19-related myopathy. Decreased muscle bulk (sarcopenia) and fatty infiltration (myosteatosis) may be due to a combination of denervation atrophy, deconditioning due to prolonged immobilization, immune-mediated myopathy, toxic or drug-related myopathy, and nutritional deficiencies.

### Mycobacterial infection

Extrapulmonary tuberculosis (TB) accounts for 15–20% of all *Mycobacterium tuberculosis* infections. As many as 1–3% of all TB cases involve the musculoskeletal system, usually due to lymph-hematogenous spread of primary pulmonary infection [38]. Musculoskeletal TB most commonly presents as tuberculous spondylitis (Pott’s disease), but peripheral arthritis, osteomyelitis, tenosynovitis, and bursitis can also occur, in decreasing order of frequency [39].

**Fig. 4:**
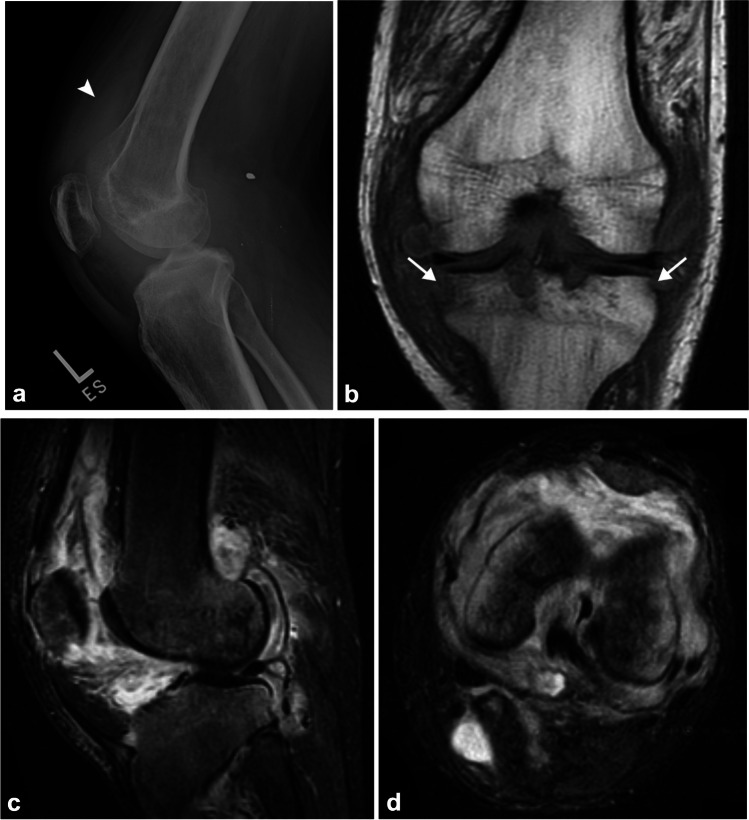
Thirty-eight-year-old man with right knee pain and swelling. Lateral radiograph (**a**) and Cor T1 (**b**), Sag STIR (**c**), and axial STIR (**d**) MR images of the right knee demonstrate juxta-articular osteopenia with a peripheral erosion (*arrow*) and relative preservation of the joint space with only gradual narrowing over a period of five months (Phemister’s triad) and a large joint effusion (*arrowhead*) with extensive synovitis on MRI. Joint aspiration demonstrated acid-fast bacilli, and cultures grew *Mycobacterium tuberculosis*, diagnostic for tuberculous arthritis

Tuberculous arthritis mainly involves weight-bearing joints such as the sacroiliac, hip, and knee joints, and is usually monoarticular (90%) [40]. The classic radiographic findings include juxta-articular osteopenia, peripheral/marginal osseous erosions, and gradual progressive joint space narrowing, known as Phemister’s triad (Fig. 4) [41]. There is gradual joint space narrowing as the synovitis with early-stage TB leaves the articular cartilage intact, although secondary degenerative change and eventually ankylosis can be seen in later stages [40]. There can also be peri-articular abscesses and soft tissue calcifications.

**Fig. 5:**
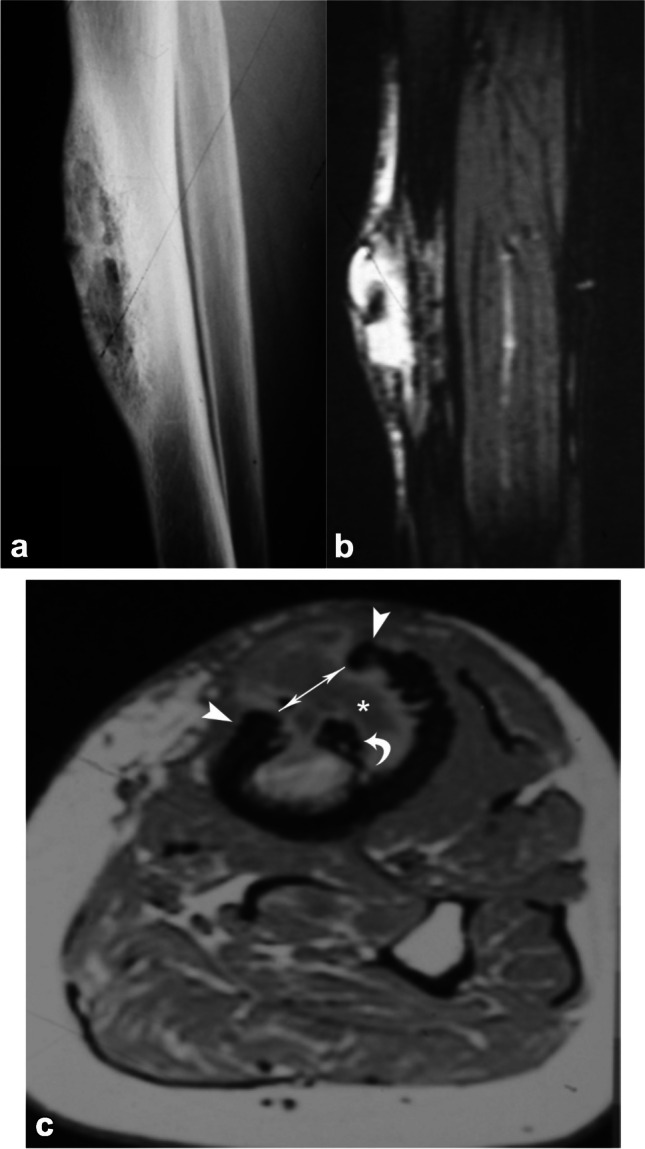
Tuberculous osteomyelitis of the tibia. Lateral radiograph (**a**) demonstrates expansile lytic lesion of the anterior cortex of the mid tibial diaphysis with corresponding fluid signal intensity and adjacent bone marrow edema on sagittal STIR MRI (**b**). Axial T1 MRI (**c**) shows intraosseous abscess (*), sequestrum (*curved arrow*), cloaca (*double headed arrow*), and involucrum (*arrowheads*) with the abscess extending into the soft tissues through the cloaca

Extraspinal tuberculous osteomyelitis typically affects the lower extremity bones (Fig. 5) [42]. Radiographs demonstrate osteopenia with metaphyseal lytic lesions which likewise show low T1 and high T2 signal intensity on MRI, with peripheral enhancement in cases of intraosseous abscesses [43, 44]. In children, a differentiating feature of tuberculous compared with pyogenic infection is spread across the physis, which may lead to balloon-like swelling and cyst-like cavity appearance of the tubular bones of the hands and feet with dactylitis, also known as spina ventosa [45]. Muscle, subcutaneous, and skin involvement in TB is rare, with psoas abscesses the most common manifestation secondary to spondylodiscitis, though notably with absence of significant surrounding edema, myositis, or cellulitis (“cold abscess”) [39, 43].

Atypical mycobacteria account for 0.5–30% of all mycobacterial infections, most commonly in elderly or immunocompromised patients, with 5–10% involving the musculoskeletal system [46]. Infection may be from direct inoculation from environmental or contiguous sources or from hematogenous spread. Musculoskeletal manifestations can be similar to TB infection, with aspiration or biopsy required for differentiation. Osseous infection is most common with *Mycobacterium kansasii* and *Mycobacterium scrofulaceum*, followed in frequency by *Mycobacterium avium-intracellulare* (MAC) and *M. fortuitum*, though it may take several weeks for osteolysis and periostitis to become radiographically evident [46, 47]. In cases of lower extremity atypical mycobacterial infection as a result of direct inoculation from minor penetrating injuries, soft tissue manifestations such as cellulitis, abscess, septic bursitis, and septic tenosynovitis are more common than with TB [46].

**Fig. 6:**
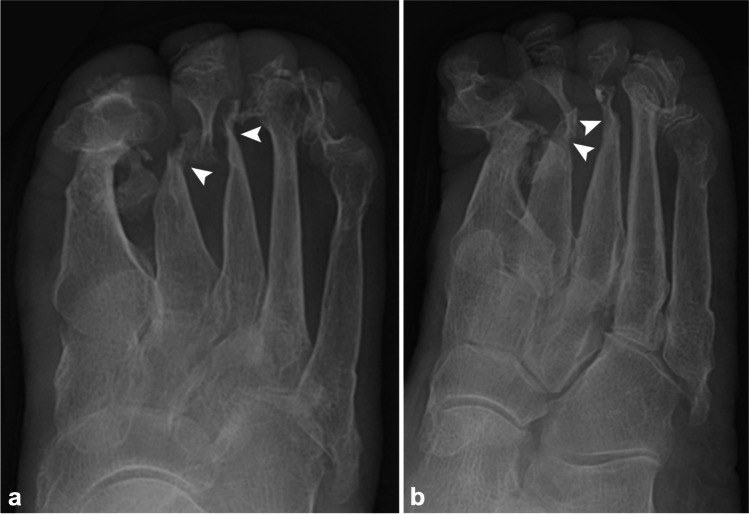
Eighty-five-year-old woman with leprosy (Hansen’s disease) and right foot deformity. AP (**a**) and oblique (**b**) radiographs demonstrate arthritis mutilans with osseous destructive changes of the distal metatarsals and phalanges with a “licked candy stick” appearance of the distal second and third metatarsals (*arrowheads*), due to leprosy induced neuropathic arthropathy

Leprosy (Hansen disease) is an infection by *Mycobacterium leprae*, which has a special predilection for the skin and nerves [48]. Untreated leprosy can lead initially to sensory loss, which increases the frequency of minor injuries and ultimately leads to infection and, eventually, mutilating injuries. On imaging, nerves affected by leprosy can appear enlarged, with loss of the normal fascicular pattern, increased flow on Doppler ultrasound, and T2 hyperintensity, and show gadolinium enhancement on MRI. US and MRI have reported sensitivities of 74% and 92%, respectively, with active disease [49]. Due to this enlargement, nerve compression in fibro-osseous tunnels can be seen in up to a third of cases. Up to 60% may have joint symptoms, including pain and swelling, in a pattern that may mimic rheumatoid arthritis [50]. Chronic infection may lead to neuropathic arthropathy in the feet, with four main types affecting the (1) ankle; (2) midtarsal (Chopart); (3) tarsometatarsal (Lisfranc), or (4) subtalar joints [51]. In advanced cases, tapering of the metatarsals or phalanges can lead to an arthritis mutilans with “licked candy stick” appearance (Fig. 6), as can also be seen with psoriatic arthritis [52].

### Madura foot

**Fig. 7:**
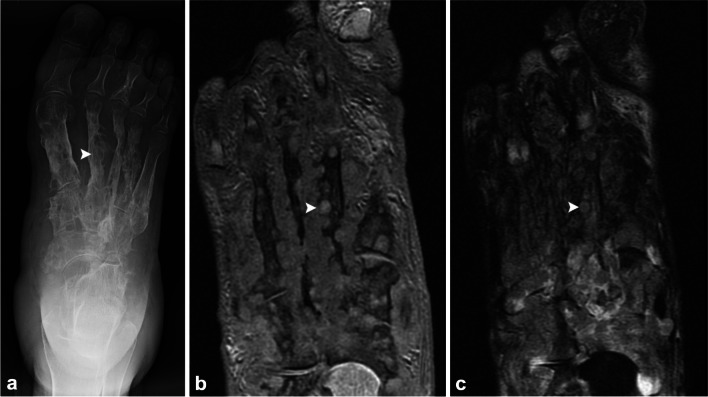
Sixty-year-old woman with chronic right foot pain and infection. AP radiograph (**a**) and long axis T1 (**b**) and STIR (**c**) MRI images of the right foot demonstrate extensive rounded cystic lucencies corresponding to T1 and T2 hyperintense mycetomas with a central T2 hypointense focus (*arrowheads* highlight representative lesion of the second metatarsal), compatible with the “dot-in-circle” sign in this patient with fungal osteomyelitis (Madura foot)

Madura foot or pedal mycetoma is a rare tropical foot infection caused by filamentous bacteria (e.g., *Actinomyces* or *Nocardia* species) or true fungi (*Madurella* species). It occurs due to direct inoculation from organisms in the soil and classically presents with hard woody swellings, discharging sinuses, and presence of organism-containing grains (mycetomas) that can progress to muscle and bone involvement with permanent disability [53]. On radiographs, foot involvement can progress through a series of findings: stage 0 = soft-tissue swelling at site of entry without bone involvement; stage I = expanding granuloma with displacement or scalloping of bone; stage II = bone irritation with periosteal reaction or reactive sclerosis; stage III = erosion or cavitation of a single bone; stage IV = longitudinal spread with joint involvement along a single ray; stage V = horizontal spread with invasion of adjacent structure of the hindfoot, midfoot, or forefoot; and stage VI = multidirectional spread with total disruption of multiple rays and rows [54]. On MRI, the characteristic finding is a pedal mycetoma, which is a microabscess containing aggregates of the organism, also known as grains or sulfur granules, leading to the “dot-in-circle” sign of multiple small (2–5 mm), round hyperintense lesions with a hypointense fibrous rim and a central low-signal intensity focus (Fig. 7) [55, 56].

### Fungal infection

Invasive fungal infections are rising due to increased number of immunocompromised patients, including those with neutropenia, human immunodeficiency virus (HIV), chronic immunosuppression, indwelling prostheses, burns, diabetes mellitus, and those on broad-spectrum antibiotics [57]. Fungal infections typically occur in endemic regions and may spread by direct skin inoculation or hematogenously, usually via the lungs [58]. Fungal infections may present with soft tissue nodules (granulomas), sinus tracts to the skin, multifocal chronic osteomyelitis, and chronic granulomatous joint involvement, with imaging findings overlapping with mycobacterial infection.

The musculoskeletal system is the fourth most common system involved with Aspergillus (*Aspergillus fumigatus*) infection due to hematogenous spread from invasive pulmonary infection, which can cause osteomyelitis with multifocal lytic lesions, often of the pelvis and knee. Blastomycosis (*Blastomyces dermatitidis*) is usually due to skin inoculate of the soles of the feet leading to subcutaneous granulomas and skin sinuses and may cause lower limb osteomyelitis and joint involvement in up to 30% of cases. Infection with Candida species in immunosuppressed patients can lead to blood-borne spread and cause osteomyelitis with osteolytic lesions of larger joints including the hip and knee, though notably without concomitant periostitis or septic arthritis.

**Fig. 8:**
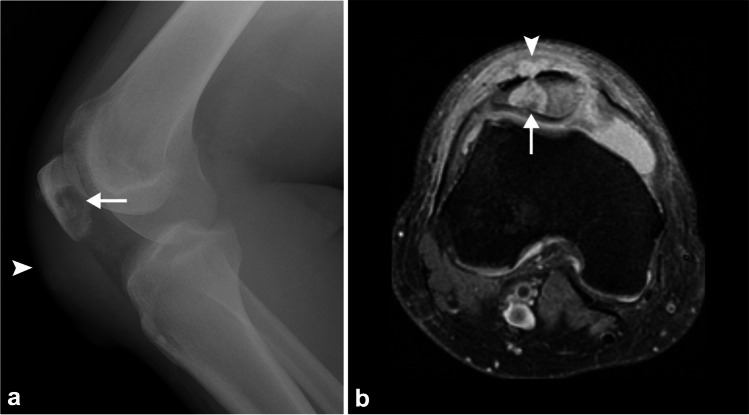
Nineteen-year-old man with disseminated coccidioidomycosis and right knee pain. Lateral radiograph (**a**) and axial PD FS MR image (**b**) of the right knee demonstrate a lytic T2 hyperintense lesion of the patella (*arrows*) with anterior cortical breakthrough and a soft tissue component with prepatellar soft tissue swelling (*arrowheads*). This patient had a confirmed histologic diagnosis of *Coccidioides immitis* osteomyelitis

Coccidiomycosis (*Coccidioides immitis*) is endemic to the southwestern USA and parts of Mexico and Central and South America, particularly the San Joaquin Valley of California (Valley fever) [59]. Musculoskeletal manifestations of coccidioidomycosis are present in 20–50% of cases with systemic spread from the lungs (1–5% incidence), most commonly resulting in axial skeletal followed by joint involvement with chronic granulomatous synovitis and periarticular bone destruction. Osteomyelitis can also affect the lower extremities and usually presents with destructive lytic bone lesions that may be well circumscribed (punched out) in more than 50% of cases, but permeative or moth-eaten borders with loss of subchondral bone and damage to articular cartilage may also be seen (Fig. 8) [60, 61]. Although a predilection for bony prominences has been postulated, this has not been supported by subsequent literature.

**Fig. 9:**
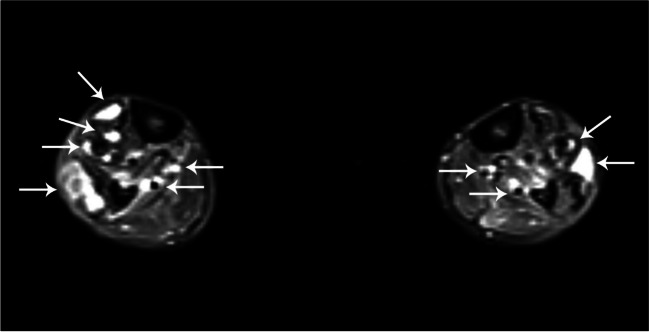
Sixty-three-year-old man with HIV and disseminated cryptococcus. Axial STIR MR image demonstrates multifocal soft tissues abscesses of the bilateral lower legs (*arrows*)

Cryptococcosis (*Cryptococcus neoformans*) is usually an opportunistic infection in AIDS patients with CD4 T cell lymphocyte counts less than 50 cells/mm^3^. Hematogenous spread from pulmonary infections can lead to discitis/osteomyelitis, multifocal osteolytic lesions, abscesses, and large joint septic arthritis, including the hips, knees, and ankles (Fig. 9) [58]. Sporotrichosis (*Sporothrix schenckii*) is the most common fungal infection of the deep soft tissues of the extremities and is usually transmitted via contaminated wood (thorns) in the hands and feet of agricultural workers and gardeners. Bone involvement can be proliferative and sclerotic. Histoplasmosis (Histoplasma species) is endemic to the Ohio and Mississippi river valleys and most often causes pulmonary granuloma formation that resembles tuberculosis, though hematogenous spread can lead to osteomyelitis, with a focal osteolytic lesion of long bones that can be confused with bone tumors [62].

### Parasitic infection

Although parasitic infection is common worldwide, often as a consequence of poor sanitation (untreated drinking water), musculoskeletal involvement is uncommon compared to visceral involvement [30]. Cutaneous manifestations such as subcutaneous cystic nodules (cysticercosis), skin ulcers (dracunculosis), localized subcutaneous (Calabar) swellings (loiasis), fibrous nodules (onchocerciasis), pruritic papular dermatitis (schistosomiasis), eyelid edema, and subungual hemorrhage (trichinosis) are generally diagnosed clinically, with imaging typically not indicated. Among parasitic infections, helminthic infections most often cause musculoskeletal manifestations, and can be divided into nematodes (worms), trematodes (flukes), and cestodes (tapeworms).

Nematodes may be divided into intestinal and tissue worms, where the former does not but the latter can involve the musculoskeletal system. Trichinosis (*Trichinella spiralis*) is usually caused by ingestion of raw or inadequately cooked pork containing encysted larvae, which disseminate through the body. Those that reach skeletal muscle and survive can cause myositis. Filariasis is caused by infection with *Wuchereria bancrofti* or less commonly *Brugia malayi* spread by mosquitoes. Once inoculated, larval worms mature and grow into adult worms that colonize the lymphatics and can lead to marked swelling of the lower extremities, causing lymphangitis and ultimately elephantiasis. This manifests as blurring of the subcutaneous fat planes with a linear striated pattern and soft tissue calcification on plain radiographs [30, 63]. Other filarial infections such as onchocerciasis, loiasis, and dracunculosis are caused by larger worms that do not obstruct lymphatics and more commonly calcify, with alive subcutaneous worms occasionally detected by ultrasound. Migration of worms near a joint may cause a chemical synovitis.

**Fig. 10:**
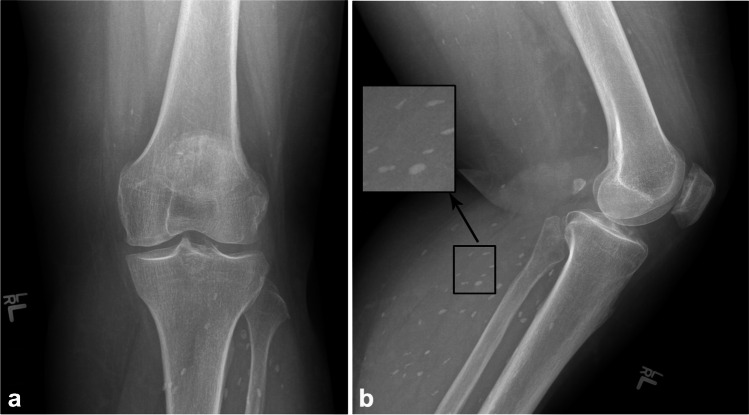
Sixty-year-old woman with left knee pain. AP (**a**) and lateral (**b**) radiographs demonstrate numerous intramuscular “rice grain” calcifications, compatible with cysticercosis from prior ingestion of *Taenia solium* eggs via fecal–oral transmission

**Fig. 11:**
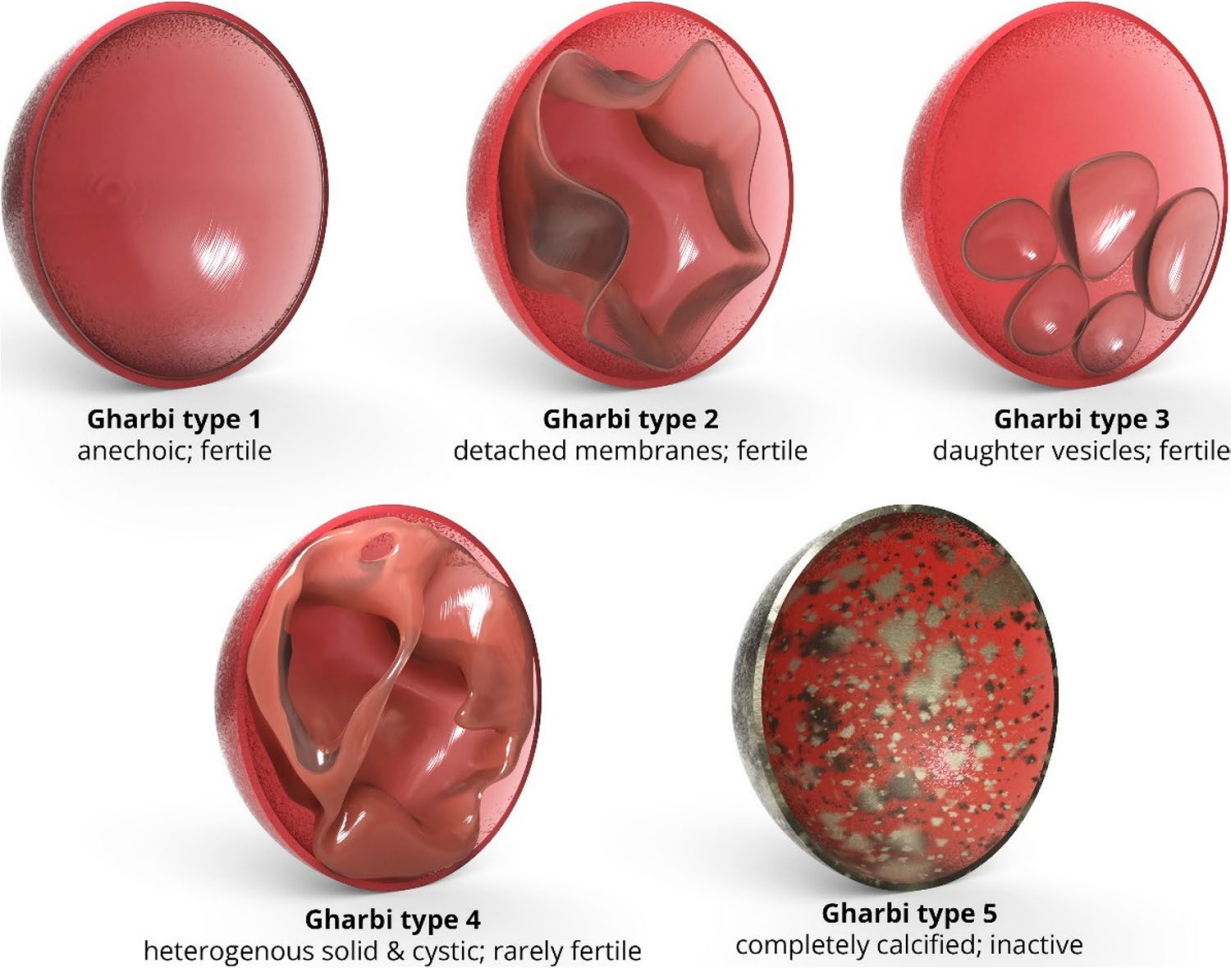
Illustration demonstrating the Gharbi classification of echinococcal cyst types and appearances

**Fig. 12:**
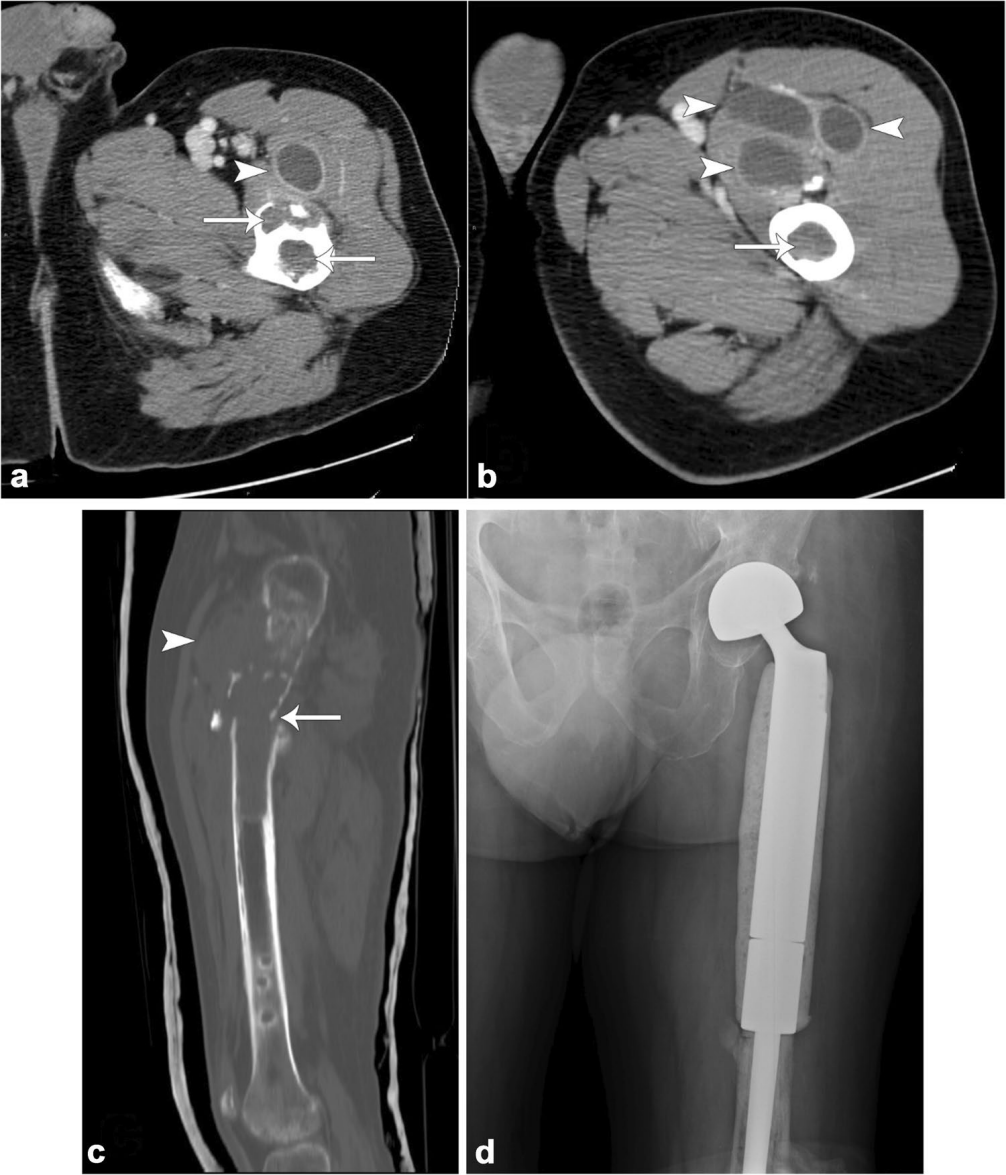
Sixty-year-old male farmer and shepherd from Southern Italy with left femur echinococcosis with pathological fracture. Axial (**a**, **b**) and sagittal (**c**) CT images demonstrate a 15-cm lytic lesion of the proximal femur with pathological fracture (*arrows*) and multilocular daughter cysts (*arrowheads*) extending into the vastus intermedius muscle. AP radiograph (**d**) 20 years later shows that the patient has done well after proximal femur resection and left total hip modular endoprosthesis placement

Cestodes (tapeworms) infection is most commonly due to fecal–oral transmission by cats or dogs or through improperly cooked meat. Cysticercosis (*Taenia solium*) can penetrate the intestinal wall and travel to the muscles to form palpable cysts and elongated “rice grain” calcifications (Fig. 10) [30, 63]. Hydatid disease is most commonly caused by the larval stage of *Echinococcosis granulosus* [64]. Although it may occur almost anywhere in the body, its most frequent targets include the liver (65–70%) and lungs (25–30%). Muscle (0.5–4%) and bone (0.5–2.5%) involvement is rare. Muscular involvement most commonly affects the paravertebral, gluteal, and lower extremity muscles. On cross-sectional imaging, typical features include multivesicular cysts with intracystic membranes (daughter vesicles or cysts). Curvilinear or ringlike calcifications are seen in 20–30% of cases [65]. There are five types of appearances by the Gharbi classification: type I: simple cyst with laminar membrane visible (snowflake sign; fertile cyst); type II: detached membrane (serpent sign; fertile cyst); type III: cyst with multiple daughter cysts (spoke wheel or honeycomb pattern; usually fertile cyst); type IV: heterogenous lesion solid and cystic (rarely fertile); and type V: calcified cyst (inactive) (Fig. 11) [66]. The World Health Organization (WHO) classification also adds a type CL for a non-specific cystic appearance without visible membranes that can occur in up to 25% of cases of hydatid cysts. Collapse can result in the “water-lily sign” with a floating membrane appearance. Free-floating brood capsules with white sediment (“hydatid sand sign”) and “fluid–fluid levels” may also be seen [63]. Hydatid disease of the bone most commonly occurs in well-vascularized areas such as vertebrae and the long bones, with 50% occurring within the spine, followed by the pelvis, femur, and tibia. On radiographs, there is osteolytic change that can progress to fill and eventually expand the medullary cavity and may mimic an aneurysmal bone cyst, giant cell tumor, myeloma, or cystic metastasis (Fig. 12). Findings may progress to erosion through the cortex with involvement of the adjacent soft tissues, eventually leading to pathological fracture. Periosteal reaction or sclerosis are uncommon. Extraosseous hydatid cysts may calcify, but intraosseous cysts rarely do. Biopsy and aspiration are generally contraindicated due to risk of spillage, which may trigger anaphylaxis.

Most trematodes (flukes) preferentially involve the abdominal organs. Freshwater snails are intermediate hosts. Bathing or wading in infested waters allows the parasite to burrow under the skin, where it then migrates to and matures within the liver and can migrate and can subsequently lodge in the venules of the bowel and genitourinary system. Rarely, schistosomiasis (bilharziasis) has been reported to cause polyarthropathy due to joint involvement.

Protozoan infections rarely cause musculoskeletal manifestations. Toxoplasmosis (*Toxoplasma gondii*) can occasionally cause osseous lesions, with metaphyseal alteration of tubular bones that can simulate rubella [63]. Giardiasis (*Giardia lamblia*), also a gastrointestinal protozoan, has been reported to cause reactive arthritis, including sacroiliitis and synovitis. Protozoan infections such as malaria and kala azar (*Leishmania donovani*) infection can cause myalgia; however, imaging is generally not useful [30].

## Selected potential mimics

**Fig. 13:**
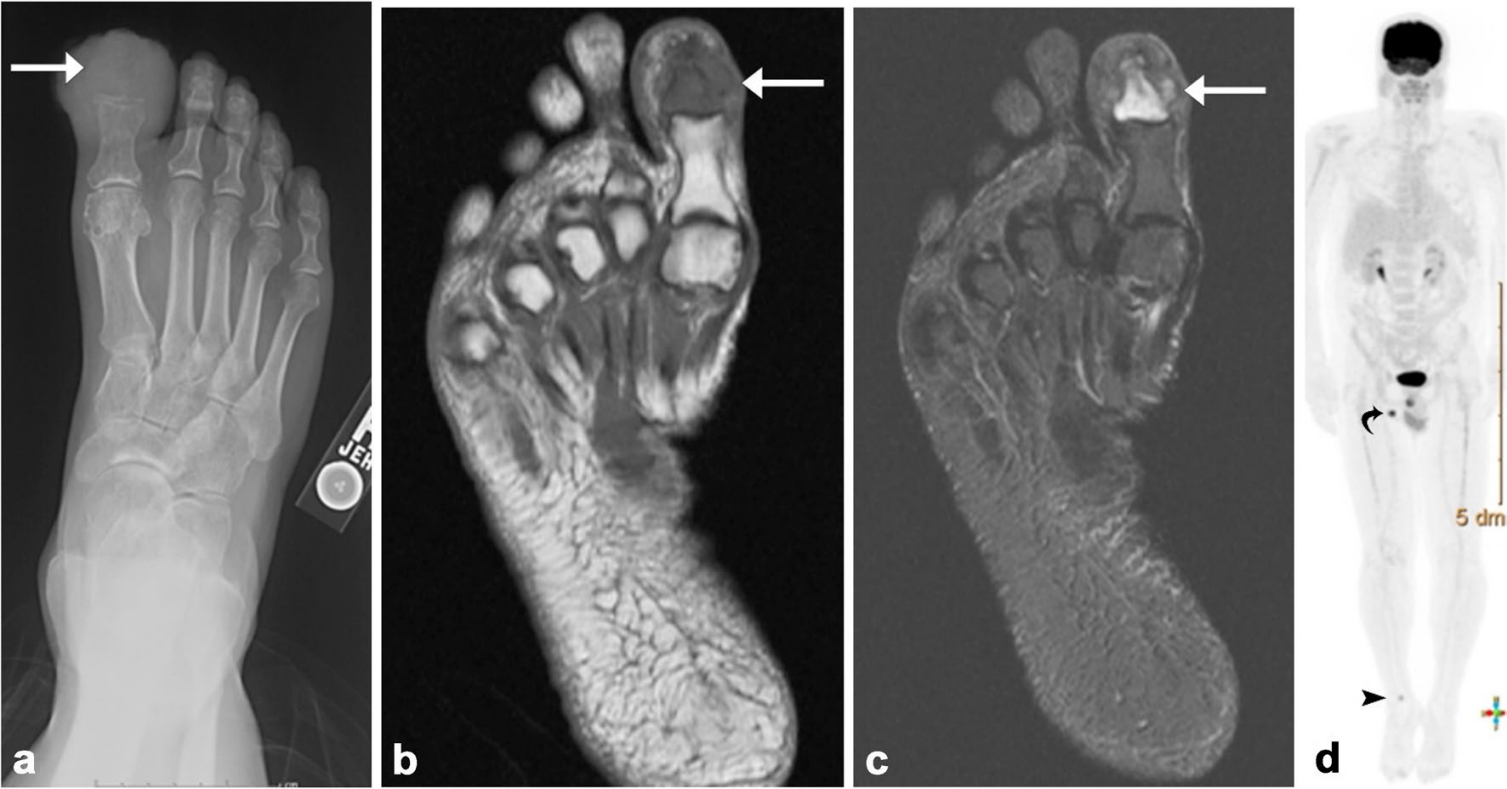
Osteosarcoma mimicking osteomyelitis. Fifty-eight-year-old male with right great toe pain. AP radiograph of the right foot (**a**) demonstrates an aggressive lytic lesion of the first distal phalanx (*arrow*). MR examination of the foot reveals a marrow-replacing lesion in the first distal phalanx (*arrows*), demonstrating T1 hypointensity (**b**) and T2 hyperintensity (**c**), raising suspicion for osteomyelitis. However, subsequent tissue sampling confirmed the lesion as osteosarcoma. Restaging whole body ^18^F-FDG PET-CT (**d**) after surgical resection and multiple cycles of chemotherapy shows hypermetabolic activity of the right ankle (*arrowhead*) and inguinal lymph nodes (*curved arrow*), with respective SUVmax values of 4.4 and 10.4, indicative of metastatic lymphadenopathy

There are several potential mimics of lower extremity infections that should be considered when evaluating patients with suspected infections, including chronic nonbacterial osteomyelitis (CNO), foreign body granuloma, and inflammatory arthropathies such as gout, lymphedema, and Morel Lavallée lesions (MLLs). These conditions are characterized by swelling and erythema, along with fever and elevated inflammatory markers (WBC and CRP). In order to prevent misdiagnosis, it is essential to assess imaging findings alongside clinical information, demographics, and laboratory test results (Fig. 13).

### CNO/CMRO

Chronic nonbacterial osteomyelitis (CNO) is a relatively rare autoinflammatory disease occurring primarily in children and adolescents. It is a non-infectious disorder causing recurrent musculoskeletal pain. Chronic recurrent multifocal osteomyelitis (CRMO) is a particularly severe manifestation of CNO, typically distinguished by symmetric inflammatory bone lesions and a waxing and waning disease course [67]. The characteristic CNO/CMRO lesions are multiple and symmetric, usually affecting metaphyses of long bones of the lower extremities near the knees and ankles. There is currently no definitive diagnostic test to confirm the presence of CNO. Typically, CNO is a diagnosis of exclusion by ruling out other diseases such as acute bacterial osteomyelitis, bone tumors, and blood disorders. Distinguishing between CNO and extremity infection can be challenging as they can present with similar symptoms such as localized pain, swelling, and erythema. Imaging plays a crucial role in the diagnosis, differential diagnosis, and monitoring of CNO patients.

#### Radiography

Plain radiographs are commonly utilized as the first step in screening and to exclude fractures in CNO patients. Radiographically, findings may manifest as lytic, sclerotic, or mixed bone lesions, with hyperostosis and periosteal reaction. However, the lack of these findings does not necessarily rule out CNO [68].

#### Nuclear medicine

Whole body bone scintigraphy is a useful imaging technique for the diagnosis and monitoring of CNO. It can detect clinically silent lesions and monitor disease progression. However, novel more sensitive imaging modalities, such as WB-MR, have largely replaced bone scintigraphy due to their greater accuracy and the absence of radiation exposure. As such, bone scintigraphy should only be considered when WB-MRI or serial MRIs are not available.

#### CT

While CT is not typically recommended for children with suspected CNO given the associated radiation exposure and advantages of MRI, there may be certain cases where it could be useful. For example, in scenarios where MRI is unavailable or when there is persistent doubt even after MRI, CT can serve as a valuable diagnostic aid [69].

#### MRI

Currently, WB-MRI is the most sensitive and preferred imaging modality for the diagnosis and monitoring of CNO. WB-MRI is recommended for screening silent lesions as it can identify asymptomatic and radiographically hidden multifocal lesions, all while sparing children from radiation. Additionally, it provides information on lesion distribution and soft tissue involvement, making it a useful tool for excluding differential diagnoses including infection [70].

**Fig. 14:**
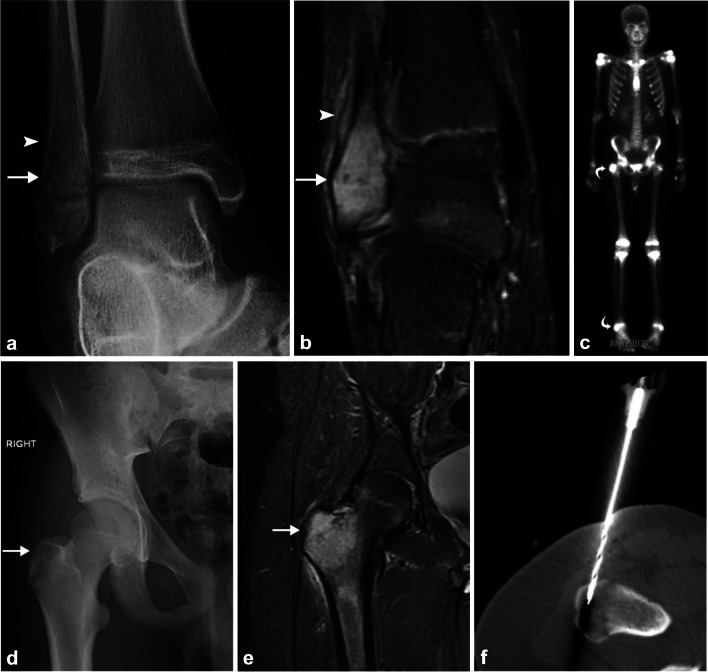
Chronic nonbacterial osteomyelitis (CNO) mimicking bacterial osteomyelitis. Fourteen-year-old male with right hip and ankle pain. AP radiograph of the right ankle (**a**) reveals permeative lucency (*arrow*) of the lateral malleolus with periosteal reaction (*arrowhead*) along the distal metadiaphysis. Coronal STIR MRI image (**b**) exhibits edema (*arrow*) and periostitis (*arrowhead*). Tc^99m^-MDP bone scan (**c**) demonstrated increased uptake in the right lateral malleolus and also the right greater trochanter (*curved arrows*). AP radiograph of the right hip (**d**) also shows permeative lucency of the greater trochanter (arrow) with corresponding bone marrow (*arrow*) and adjacent soft tissue edema on coronal STIR MRI (**e**). The diagnosis of chronic nonbacterial osteomyelitis was confirmed by bone biopsy of the greater trochanter (**f**) with histopathological analysis revealing nonspecific inflammatory changes and negative culture results

Overall, a combination of imaging findings (mainly using WB-MRI), clinical presentation, and laboratory parameters are required to differentiate CNO from extremity infection (Fig. 14).

### Foreign body granuloma

Foreign body granuloma can arise either from an iatrogenic gossypiboma, caused by the retention of surgical sponges or instruments during an operation, or from a penetrating foreign body that results in the formation of granulation tissue around it. On occasion, foreign body granulomas and infections in the lower extremities can present with similar clinical symptoms. More importantly, infection, including cellulitis and soft tissue abscesses, is the most frequent complication associated with retained foreign bodies. Therefore, it is imperative for radiologists to be familiar with both conditions for early diagnosis and effective management.

#### Radiography

Conventional radiography serves as an initial screening tool for suspected foreign body cases, with a detection rate of 80% for all foreign bodies [71]. If the foreign body is radiopaque, it is often easily identifiable. However, it may be challenging to identify radiolucent foreign bodies like wooden splinters using plain radiography [72].

#### Ultrasound

**Fig. 15:**
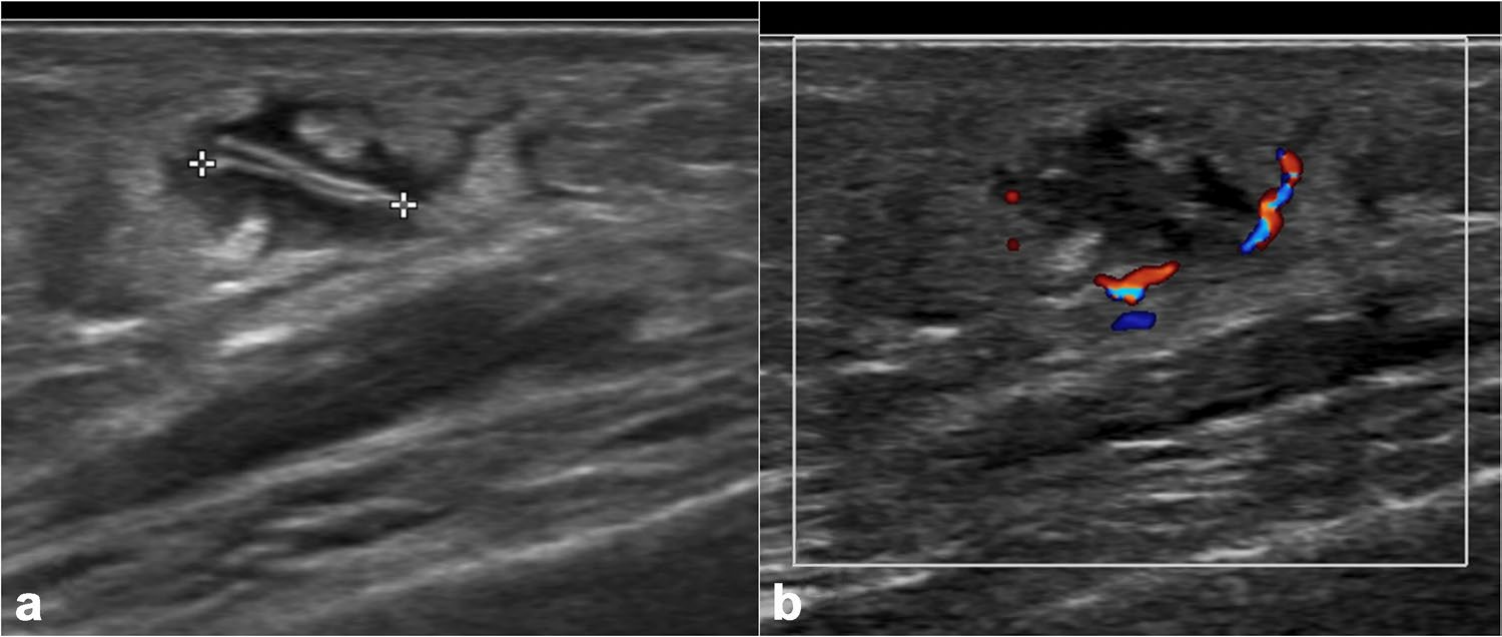
Fourteen-year-old boy with left thigh subcutaneous foreign body granuloma after accident. Grayscale ultrasound (**a**) demonstrates an 8-mm linear hyperechoic foreign body with surrounding hypoechoic foreign body granuloma and surrounding hyperemia on color Doppler image (**b**). The patient was asymptomatic and not removed; thus, the exact nature of this foreign body is unknown

Ultrasound may also be useful for identifying foreign bodies in cases of clinical concern. Sonography is both sensitive and specific in detecting and localizing foreign bodies. While many foreign bodies are radiolucent and may remain undetectable on plain radiography, all of them are hyperechoic on sonography and will demonstrate posterior acoustic shadowing [73]. They are typically surrounded by hypoechoic halos consisting of reactive lesions such as hematoma, edema, and granulation tissue (Fig. 15) [74]. Additionally, ultrasound can identify foreign body complications including infection [75]. Furthermore, it can offer immediate imaging guidance for the extraction. Thus, when evaluating suspected radiolucent foreign bodies, ultrasound is considered the most effective imaging technique.

#### CT

CT is an effective method for suspected foreign objects that were not identified through radiography or ultrasound, particularly in the deeper compartments. CT is 5–15 times more sensitive than radiography and can even detect radiolucent objects such as plastic and wood [76]. A high specificity of 98% and moderate sensitivity of 68% has been suggested for CT in detecting foreign bodies of all types [77]. The appearance of foreign bodies on CT scans can vary, with acute cases showing a low-density area and chronically retained cases showing a high-density area [78].

#### MRI

On both T1- and T2-weighted images, foreign bodies appear as low signal or signal void to the muscles [79]. Metallic foreign bodies may demonstrate prominent blooming (susceptibility) artifact, especially on gradient echo or susceptibility-weighted imaging. However, identification of other small foreign bodies on MRI can be challenging. The main advantage of MRI over other imaging methods is superior soft tissue contrast, which can be helpful for identifying complications including infection. Thus, when infections are suspected, MRI is the method of choice. MRI also delineates soft tissue abscesses as thick-walled rim enhancing fluid collections, possibly with septations and internal debris, and will show restricted diffusion on DWI. It can also detect osteomyelitis with a sensitivity and specificity of 95% and 91%, respectively, which is classically manifested with bone marrow T2 hyperintensity and corresponding T1 hypointensity [80].

In summary, when foreign body granulomas are suspected, radiologic imaging such as X-rays, ultrasound, or MRI may show the presence of a foreign body. Ultrasound is useful for detecting retained material not seen by conventional radiography, especially for superficial foreign bodies. For deeper objects, CT or MRI may be necessary, with MRI preferred for accurate visualization of soft tissue and osseous complications [29]. Depending on its age and composition, the foreign body can appear as a well-defined or irregular-shaped object with surrounding inflammatory changes, such as edema and granuloma formation. Identification of low signal or signal void foreign bodies with a characteristic ring-like reactive lesion on MRI and hyperechoic lesions with posterior acoustic shadowing on sonography is important for correct diagnosis. To avoid preventable morbidities related to FBs, it is important to integrate the radiological findings with clinical history and physical examination to arrive at an accurate diagnosis.

### Gout

**Fig. 16:**
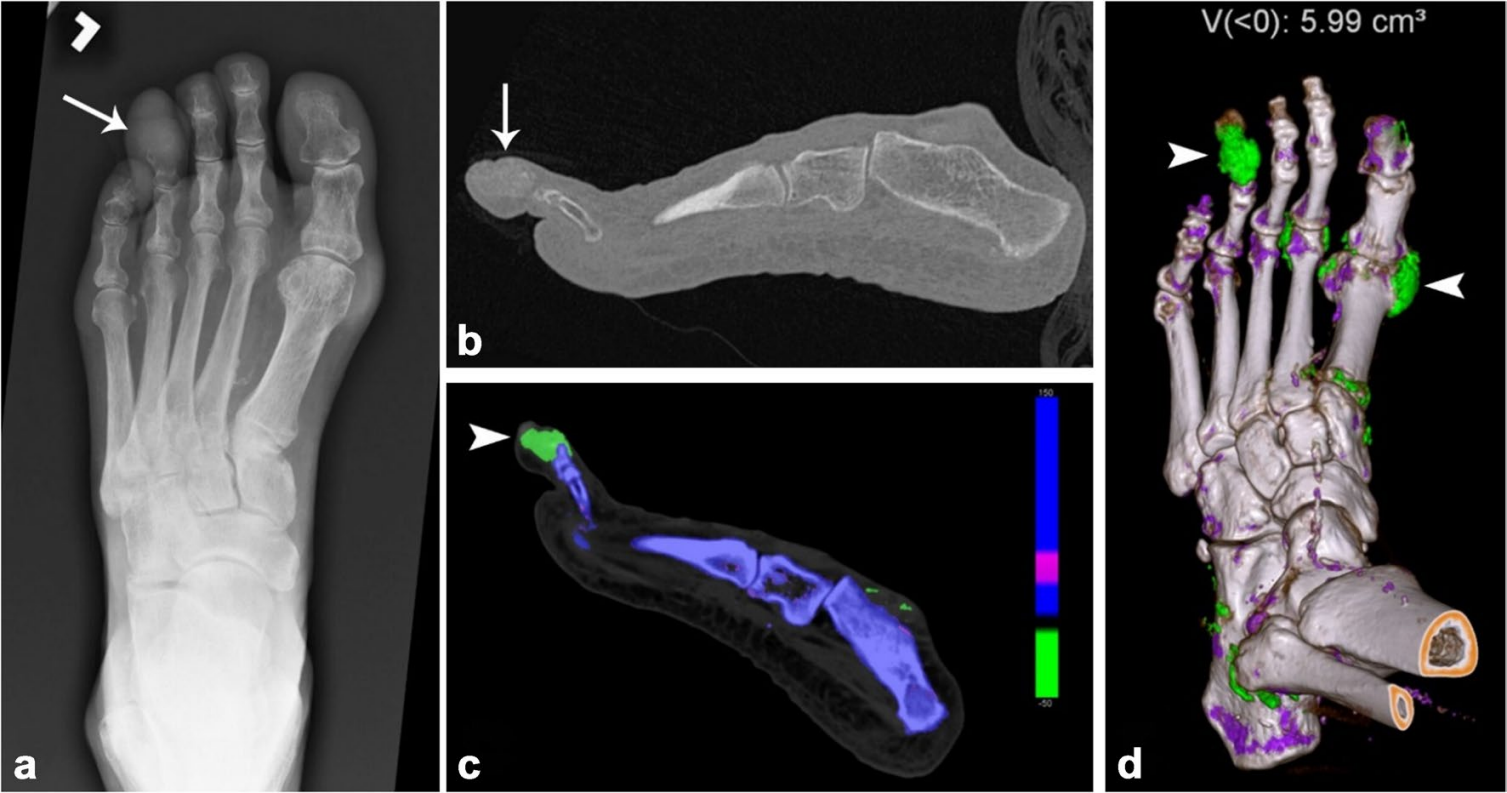
Eighty-five-year-old man with left fourth toe pain and swelling. AP radiograph (**a**) of the left foot demonstrates osseous destructive change of the fourth middle and distal phalanges (*arrow*), which could be mistaken for osteomyelitis. Dual-energy CT sagittal reformation (**b**), color map (**c**), and color-coded 3D reconstruction (**d**) demonstrate the gout (green, *arrowheads*) deposition, including intraosseous gout corresponding to the lytic areas (*arrow*). The calculated volume of monosodium urate deposition of the left foot was 5.99 cm.^3^

Gout and infection are the main differentials in patients presenting with acute monoarthritis [81]. Failure to distinguish between these two diseases could lead to undesirable outcomes, such as joint damage or unnecessary surgical interventions. Clinical differentiation between acute gout and septic arthritis is challenging, and there may be cases where the two conditions coexist. Intraosseous gout can also mimic osteomyelitis (Fig. 16). Synovial fluid analysis is the preferred diagnostic tool for both conditions, which involves identifying monosodium urate crystals with a polarized microscope for gout and bacteria through gram staining or culture for septic arthritis. However, synovial fluid analysis is often impractical in clinical practice, and distinguishing between gout and septic arthritis remains challenging. In this setting, imaging may play an important role alongside the clinical and laboratory findings.

Radiography is useful in revealing the classic punched-out erosions with overhanging edges and tophi formation in chronic cases, as well as joint erosions and osseous destruction in recurrent episodes. Therefore, x-rays are frequently essential, and it may be the only necessary imaging modality for diagnosis. However, at initial acute presentation, radiography may only display non-specific soft tissue swelling, which can make diagnosis and management challenging. In this scenario, the use of more advanced imaging techniques, such as MRI, has the potential to serve as a valuable problem-solving tool. MRI findings can appear similar to those of infection, including joint effusion and soft tissue fluid collections with thick rim enhancement, as well as marrow edema and enhancement. However, the presence of gouty tophi can serve as a useful differentiating factor, which are typically low signal intensity on both T1- and T2-weighted images [82]. Dual-energy CT (DECT) has high diagnostic accuracy for the diagnosis of gout, with a sensitivity of 88% and specificity of 90% [83]. DECT material decomposition algorithms can be used to quantify the amount of monosodium urate deposition and follow-up treatment response [84]. Nevertheless, despite the importance of imaging, definitive diagnosis should be based on joint aspiration and synovial fluid analysis when there is clinical suspicion of septic arthritis or if a diagnosis of gout has not been conclusively established [85, 86].

### Other inflammatory arthropathies

**Fig. 17:**
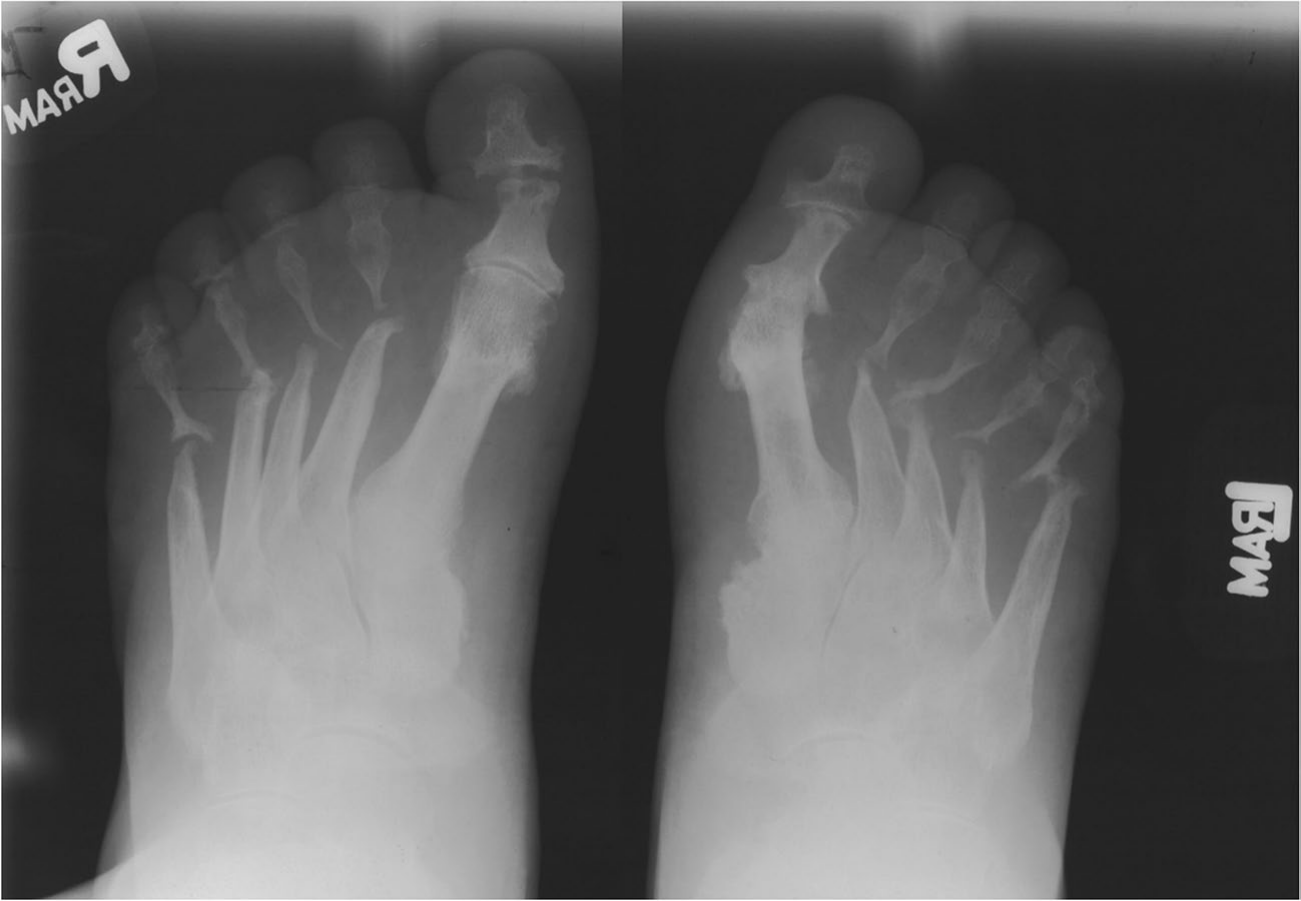
AP radiograph of the bilateral feet in a patient with psoriatic arthritis demonstrates arthritis mutilans with destructive pencil-in-cup deformities of the bilateral second through fifth metatarsophalangeal joints and fusion of the left first metatarsophalangeal and bilateral second through fifth proximal interphalangeal joints

Other types of inflammatory arthropathies like rheumatoid arthritis (RA) and psoriatic arthritis (PsA) can also resemble infection both in clinical and radiographic manifestations, necessitating clinical suspicion of these conditions. Both RA and PsA can cause synovitis, tenosynovitis, and erosive changes, which can mimic septic arthritis [87]. RA is more likely to cause juxta-articular osteopenia and alignment deformities, and PsA is more likely to cause enthesitis, periostitis, and more likely to progress to fusion or arthritis mutilans with pencil-in-cup deformities (Fig. 17) [88]. PsA can also present as a spondyloarthritis with asymmetric sacroiliitis. Like gout, subsequent joint aspiration and fluid analysis can assist in making a definite diagnosis in challenging cases.

### Lymphedema

**Fig. 18:**
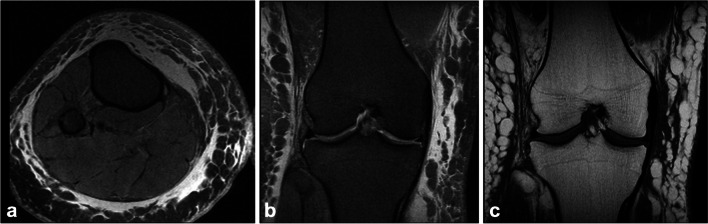
Forty-year-old woman with gradual onset of right knee pain and swelling. Axial (a) and coronal (b) PD FS and coronal T1 (c) MRI images demonstrate extensive diffuse subcutaneous edema with a clinical diagnosis of lymphedema since 13 years of age. Cellulitis and edema of other causes could have a similar appearance on MRI

Lymphedema, whether primary or secondary, is typified by chronic swelling, localized pain, and atrophic skin changes. Currently, lymphedema is usually diagnosed based on a patient’s history and physical exam findings. However, lower extremity lymphedema can frequently be mistaken for other causes of extremity edema and enlargement, including infection. Additionally, lymphedema itself is highly susceptible to breakdown and subsequent infection, which further complicates the picture [89]. It is crucial to identify infection with or without lymphedema in patients with limb swelling, as this alters management strategies and prognosis. In this regard, imaging techniques (lymphoscintigraphy, ultrasonography, CT, and MRI) can assist in identifying lymphedema (Fig. 18).

#### Lymphoscintigraphy (LSG)

**Fig. 19:**
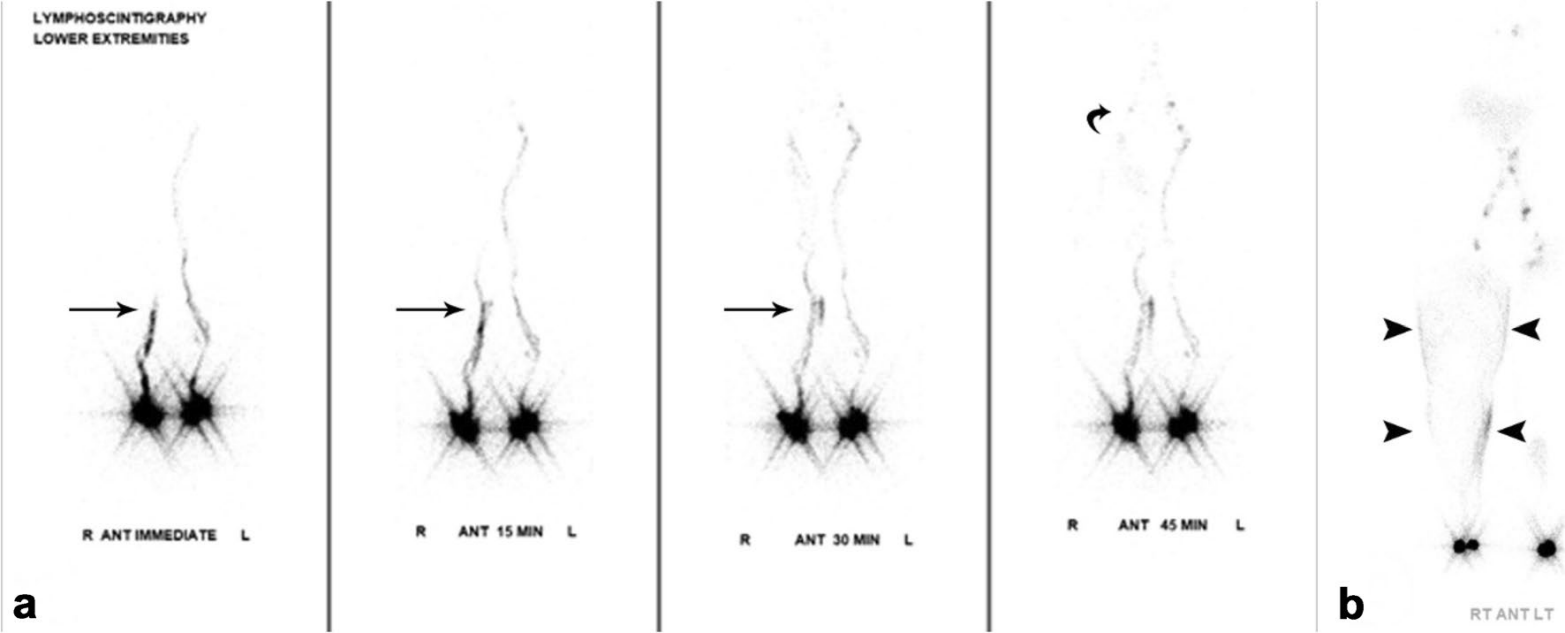
Sixty-one-year-old woman with stage IV vulvar cancer and right lower extremity lymphedema. Tc^99m^ sulfur colloid lymphoscintigraphy (**a**) shows asymmetric and delayed passage of tracer activity in the right lower extremity lymphatics (arrows) compared to the left, not reaching the right inguinal lymph node (*curved arrow*) until 40 min after injection. Delayed images (**b**) show extensive dermal backflow of the right lower extremity (*arrowheads*) throughout the thigh and lower right leg

Currently, LSG serves as the standard diagnostic test for confirming lymphedema. Abnormal LSG findings with delayed transit time of the radiolabeled colloid to regional lymph nodes, dermal backflow, asymmetric node uptake, and/or collateral lymphatic channels indicate impaired lymphatic function and aid in the diagnosis of lymphedema (Fig. 19) [90].

#### Lymphography

Lymphography involves injecting radio-opaque material directly into the peripheral lymph vessels. However, it is rarely performed due to the potential risk of vessel damage.

#### Duplex ultrasound (DUS)

DUS is usually the initial imaging test of choice to evaluate leg swelling. While it cannot image the lymphatic vasculature, the ultrasound characteristics of the edematous limb can provide valuable information regarding the underlying cause of the edema. In addition, DUS can grade the severity of lymphedema. Increased skin thickness, subcutaneous tissue thickness, and echogenicity of the fat with a blurred interface between the subcutaneous fat and skin are all indicative of lymphedema on ultrasound [91]. Lastly, ultrasound can help distinguish between cellulitis and abscess by identifying the presence or absence of a discrete organized fluid collection.

#### MRI

Magnetic resonance lymphangiography (MRL) can accurately evaluate lymphatic obstruction and lymph node involvement in patients with lymphedema by generating high-quality images of the lymphatic vessels. MRI is non-invasive, has improved sensitivity and specificity, and can detect lymphatic abnormalities in the early stages, the latter of which is crucial for effective management. MRL allows for a more accurate evaluation of lymphatic obstruction and lymph node involvement [92].

In summary, while a combination of medical history, physical examination, and diagnostic testing is necessary to accurately diagnose lower extremity lymphedema, effort should also be made to exclude infection using laboratory studies. If there is concern for both lymphedema and infection, a combination of imaging studies and clinical information should be used to arrive at an accurate diagnosis.

### Morel-Lavallée lesion

Morel Lavallée lesions (MLLs) represent a type of closed internal degloving injury characterized by the separation of the skin and subcutaneous tissue from the underlying fascia, resulting in a fluid-filled cavity. While MLLs can develop anywhere exposed to shear forces, certain regions are more susceptible, including the knee, greater trochanter, and anterolateral thigh, due to greater mobility of the dermis and subcutaneous tissues [93]. Despite being described over a century ago, MLL remains challenging to diagnose and manage due to their variable clinical presentation and potential complications, including infections that may share similar symptoms with MLL. Differential diagnosis between MLL and lower extremity infection is essential because management differs significantly. Radiological findings, which depend on the lesion’s chronology, can assist in the diagnosis of MLL, but a combination of clinical, imaging, and laboratory findings is necessary.

#### Ultrasound

US is a cost-effective method for evaluating subcutaneous or supra-fascial fluid collections, including MLL. On ultrasound, MLL appears as a well-defined, compressible, hypoechoic fluid collection with a thin, hyperechoic capsule, and a characteristic location between the subcutaneous fat and fascia [94]. Nodular echogenicity on ultrasound can aid in the identification of MLLs, indicating a disruption of subdermal fat globules and subsequent necrosis. However, despite being sensitive for detection of subdermal fluid collections and being helpful in many cases, the sonographic appearance remains relatively nonspecific, and may resemble other conditions, such as hematoma, inflammatory collection, myxoid or necrotic neoplasm, fat necrosis, or complex bursitis.

#### CT

Multidetector CT is useful for visualizing MLLs, particularly in patients with polytrauma. MLLs on CT have a characteristic subdermal location and may display internal complexity with a fluid–fluid layer. Compared to simple hematomas, MLLs have lower overall density. Internal islands of lipomatous density consistent with sheared fat globules may aid in further characterization of the lesion [95]. As the lesion evolves over time, there is a progressive encapsulation with potential marginal enhancement [96]. However, the overall diagnostic value of CT in identifying MLL lesions is limited, as it only confirms the presence of a fluid collection without providing much information about the lesion’s composition or density. Therefore, other imaging modalities may be more effective in assessing these characteristics.

#### MRI

**Fig. 20:**
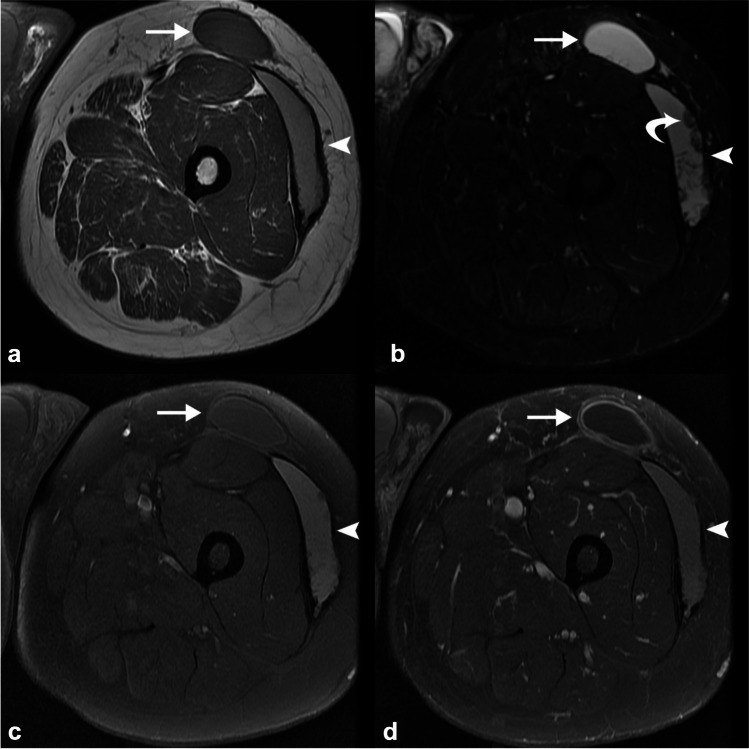
Morel-Lavallée lesions mimicking abscess and hematoma. Fifty-five-year-old man with left thigh masses and worsening pain. Axial T1 (**a**), STIR (**b**), and T1 FS pre- (**c**) and post-contrast (**d**) MRI images demonstrate two perifascial left thigh subcutaneous fluid collections. The anterior collection (*arrow*) is T1 isointense, STIR hyperintense, and has rim enhancement, which can mimic the appearance of an abscess. The lateral collection (*arrowhead*) is predominantly T1 and STIR hyperintense with a hypointense rim and areas of mural nodularity (retracted clots, *curved arrow*, **c**), which could mimic a hematoma. However, these represent type I (anterior) and type III (lateral) Morel-Lavallée lesions with a history of subacute trauma subsequently elicited

When clinical suspicion arises, MRI is the preferred imaging modality for MLL. The appearance of the lesion on MRI depends on its content and chronicity. Chronic MLL lesions are characterized by a well-defined fluid collection with a thin, hypointense capsule on T1-weighted images and hyperintense on T2-weighted images (Fig. 20) [97]. The use of fat-saturated images helps identify lipogenous foci and intralesional T1 hyperintensity. Heterogeneous T1 hyperintense areas can be seen corresponding to intracellular and extracellular methemoglobin, aiding in lesion characterization. A low T1 or T2 hemosiderin ring may become visible in conjunction with fibrous capsular formation as the lesion matures. Internal and peripheral enhancement may be noted, and areas of lipomatous signal intensity may correspond to sheared globules of subdermal fat [98, 99].

In summary, differentiating MLL from other conditions such as lower extremity infections can be challenging due to their variable clinical presentation. However, the combination of a traumatic history and the presence of a thin capsule surrounding a fluid-filled cavity in the subcutaneous tissue, along with islands of internal fat on various imaging modalities, can aid in the diagnosis of MLL. These discriminating features are important to consider for accurate identification and management of MLL.

## Conclusion

Lower extremity infection is an increasingly common cause of morbidity, and imaging plays a crucial role in evaluation. It is critical for the radiologist to be able to differentiate infection from non-infectious imaging features that can be associated with underlying conditions such as diabetes mellitus, peripheral arterial disease, and neuropathic arthropathy, and other mimics such as chronic nonbacterial osteomyelitis, foreign body granuloma, gout, inflammatory arthropathies, lymphedema, and Morel-Lavallée lesions. Although less common, it is also important for the radiologist to be familiar with the imaging manifestations of atypical infections, such as viral, mycobacterial, fungal, and parasitic infections, particularly in immunocompromised patients, those from endemic areas, or in patients with a history of travel.
